# Supplementary figures and images for: Rapid prediction of in-hospital mortality among adults with COVID-19 disease
Source: PLoS One. 2022 Jul 29;17(7):e0269813. doi: 10.1371/journal.pone.0269813 (PMC9337639; doi:10.1371/journal.pone.0269813)

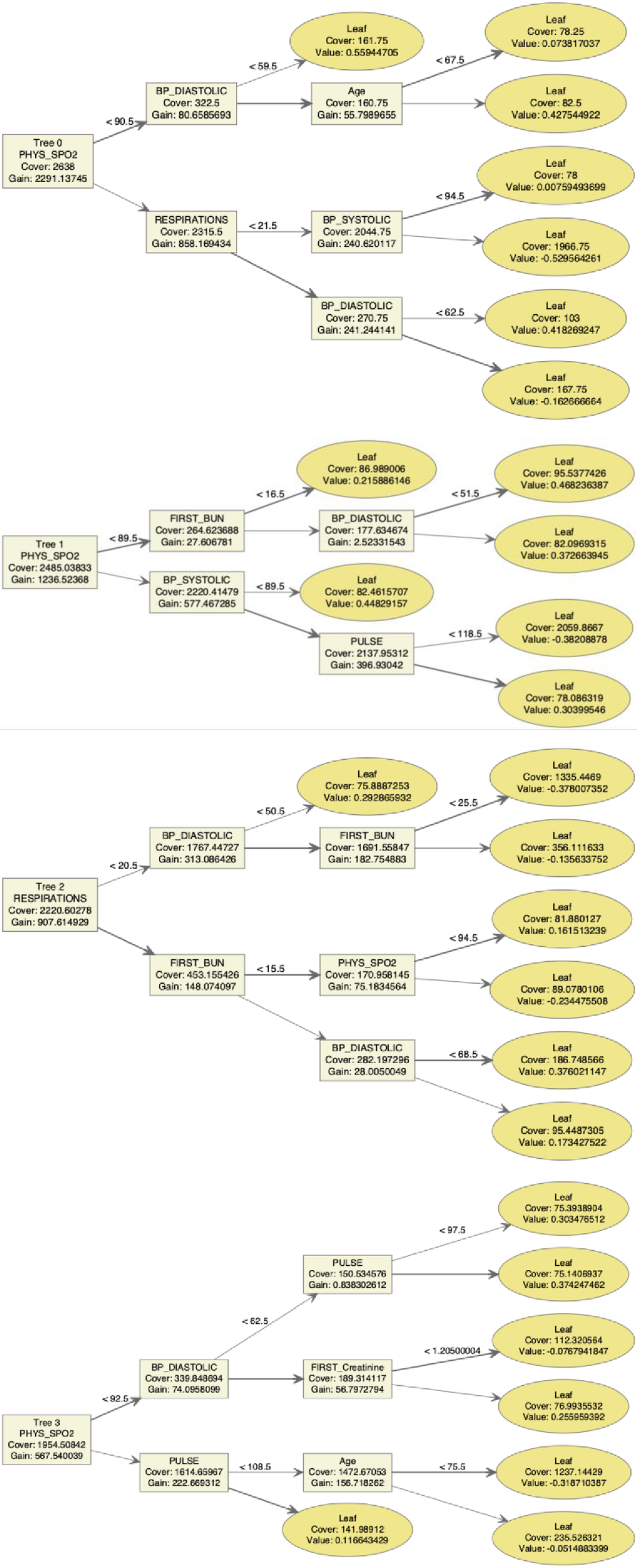

Supplement: S1 Fig — The first (top) figure (Tree 0) is the first boosted decision tree from the XGBoost model. The next (Tree 1) is the second boosted decision tree from the XGBoost model. The next tree (Tree 2) is the third boosted decision tree from the XGBoost model. The bottom tree (Tree 3) is the fourth boosted decision tree from the XGBoost model. (TIF) [file pone.0269813.s001.tif]
